# Identification of Stroke Mimics in the Emergency Department Setting

**DOI:** 10.4137/jcnsd.s2280

**Published:** 2009-03-31

**Authors:** W. Oliver Tobin, Joseph G. Hentz, Bentley J. Bobrow, Bart M. Demaerschalk

**Affiliations:** 1 Department of Neurology, Adelaide and Meath Hospital, Dublin, incorporating the National Children’s Hospital, Trinity College Dublin, Republic of Ireland.; 2 Department of Biostatistics, Mayo Clinic Arizona, U.S.A.; 3 Department of Emergency Medicine, Mayo Clinic Arizona, U.S.A.; 4 Department of Neurology, Mayo Clinic Arizona, U.S.A.

**Keywords:** stroke, transient ischemic attack, stroke mimic

## Abstract

**Background and Purpose:**

Previous studies have shown a stroke mimic rate of 9%–31%. We aimed to establish the proportion of stroke mimics amongst suspected acute strokes, to clarify the aetiology of stroke mimic and to develop a prediction model to identify stroke mimics.

**Methods:**

This was a retrospective cohort observational study. Consecutive “stroke alert” patients were identified over nine months in a primary stroke centre. 31 variables were collected. Final diagnosis was defined as “stroke” or “stroke mimic”. Multivariable regression analysis was used to define clinical predictors of stroke mimic.

**Results:**

206 patients were reviewed. 22% were classified as stroke mimics. Multivariable scoring did not help in identification of stroke mimics. 99.5% of patients had a neurological diagnosis at final diagnosis.

**Discussion:**

22% of patients with suspected acute stroke had a stroke mimic. The aetiology of stroke mimics was varied, with seizure, encephalopathy, syncope and migraine being commonest. Multivariable scoring for identification of stroke mimics is not feasible. 99.5% of patients had a neurological diagnosis. This strengthens the case for the involvement of stroke neurologists/stroke physicians in acute stroke care.

## Introduction

Thrombolysis is an established therapy for acute ischemic stroke.^1^–^3^ Administration of therapy currently requires identification of potential stroke, clinical assessment, and neuroimaging within a 270 minute treatment window.^4^,^5^ Inaccurate early diagnosis may lead to inappropriate lytic administration. Previous studies have shown a stroke mimic rate of 9%–31%,^6^–^8^ and 60% in a selected suspected TIA cohort.^9^ Our aims were to establish the proportion of stroke mimics amongst patients presenting with suspected acute stroke syndromes, to clarify the aetiology of stroke mimic and to potentially develop a clinical prediction model to aid in the identification of stroke mimics in the emergency department.

## Methods

This was a retrospective cohort observational study. Consecutive patients fulfilling stroke alert criteria were identified from July 2005 to March 2006 inclusive in a primary stroke centre serving a major metropolitan area. Study candidates were identified by a research nurse, who screened all calls to the stroke alert paging system in the primary stroke centre over the study period.

A stroke alert involved activation of an emergency stroke pager upon identification of a patient with an apparent stroke syndrome amenable to thrombolysis. This pager alerted the stroke neurologist on call, and radiology services, to the patient’s arrival. Emergency medical services (EMS) providers could trigger the stroke alert system in the field.

Stroke alert status was confirmed on arrival to Emergency Department by emergency practitioner assessment. To qualify as stroke alert, a patient must have had a known time of sudden onset of focal neurological deficit within the previous 6 hours and an abnormal Cincinnati Prehospital Stroke Scale score.^10^ The hospital was a Joint Commission certified primary stroke centre (PSC) and operated as a member of an eight PSC metropolitan matrix.^11^ The PSC was staffed 24/7 by one of 4 stroke neurologists.

Retrospective chart review of all stroke alert subjects identified the final diagnosis at discharge, along with thirty-one clinical variables. The final diagnosis was defined as the most probable diagnosis at discharge, or further follow up, either as expressed in the clinical notes by the attending stroke neurologist, or if that was not available, based on the most probable diagnosis established by consensus of a stroke neurologist and internist based on the results of neuroimaging and other investigations available. Each patient was classified as stroke or stroke mimic. Stroke patients were subclassified as ischemic or hemorrhagic. Ischemic stroke patients were further subdivided into TIA versus infarction. Hemorrhagic stroke patients were further subdivided into the various compartments of hemorrhage (epidural, subdural, intraparenchymal, intraventricular, etc.) Stroke mimics were broadly separated into the following major categories: functional, tumour, dementia, meningitis, seizure, migraine, syncope/presyncope, vestibuloneuronitis/labyrinthitis, encephalopathy, unknown and other.

The 31 variables were—Systolic and diastolic blood pressure at presentation, oxygen saturation at presentation, heart rate at presentation, presence of visual field loss at presentation, abnormal eye movements at presentation, abnormal pupillary size or reaction at presentation, blood glucose at presentation—taken either in the EMS vehicle or on arrival to the emergency department, Glasgow coma scale score at presentation taken in the EMS vehicle, presence of known cognitive impairment prior to presentation, loss of consciousness related to current presentation, known history of angina prior to presentation, initial lateralizing signs related to current presentation either on presentation to the emergency department or as witnessed by the EMS providers, atrial fibrillation on ECG either in EMS vehicle or on presentation to the emergency department, previous documented history of stroke or transient ischemic attack, known coronary artery disease, previous history of meningitis, cardiac valve prosthesis in situ, known history of seizure disorder, known history of diabetes, known history of multiple sclerosis, known history of migraine, previously diagnosed neoplasm, history of atrial fibrillation, history of hypertension, history of hyperlipidaemia, history of illicit drug use, history of renal failure, sex, age, current or past history of tobacco use and final diagnosis.

These thirty-one were arrived at using variables from previous predictive models and known causes of stroke mimic.^7^,^12^,^13^

## Statistical Anaylsis

We created a model for predicting stroke mimics by using multiple logistic regression and forward selection. We used the jackknife method to calculate the diagnostic power of the multivariable model. The jackknife method was used in order to reduce the bias that can occur when diagnostic power is calculated with the same sample that was used to create the prediction model. We performed the statistical computations by using SAS software Version 9 (SAS Institute, Cary, NC).

## Results

206 patients were reviewed. 22% of the sample was classified as a stroke mimic (see Table 1).

The presence of initial lateralizing signs was the strongest predictor of a stroke, with a positive predictive value of 90%. However the negative predictive value was only 43%.

Our multivariable model included terms for initial lateralizing signs (LAT, 0 = No, 1 = Yes), history of acute cerebrovascular event (CVE), i.e. stroke or TIA (0 = No, 1 = Yes), and diastolic blood pressure (DBP). Higher Mimic Scores indicated a higher likelihood of being a stroke mimic.

Mimic Score=0.459-1.971 LAT+0.928 CVE-0.025 DBP

The intercept was shifted so that a cut-off score of zero yielded the highest likelihood ratio. 75% of patients with a score > 0 were stroke mimics, while only 21% of patients with a score ≤ 0 were stroke mimics. The sensitivity and specificity were 7% and 99%. So, 1 of every 100 patients with stroke was misclassified as a mimic.

However, in order to have a positive score, a patient would have to have no initial lateralizing signs, a history of stroke or TIA, and a diastolic blood pressure <55 mmHg. Only 7% of the mimics, and 2% of the cohort, met these criteria.

A patient with a history of Stroke or TIA was more likely to be a stroke mimic. A patient with higher diastolic blood pressure was less likely to be a stroke mimic.

The Mimic Score, as proposed is not clinically useful.

## Discussion

The proportion of acute stroke syndrome presentations that were non-stroke was 22% in this cohort, which agrees with previous estimates.^6^,^7^,^14^

Although the mimic score is not clinically useful, it does raise some interesting questions about the aetiology of stroke mimics. The presence of lateralizing signs has previously been shown to correlate strongly with a diagnosis of stroke^15^ and hypotension, especially to diastolic values of less the 55 mmHg, would intuitively point towards a diagnosis other than stroke. However a previous history of Stroke or TIA in this model appears to increase the chance of a stroke mimic. This is presumably due to an extension of a metabolic dysfunction around a prior Stroke which exacerbated the stroke phenotype. This may occur in the setting of a metabolic derangement, infection, or hypnotic/sedating medications which would cause global cerebral dysfunction in a patient with an abnormal brain.

The commonest causes of stroke mimic in this study were seizure, encephalopathy, syncope and migraine. Seizure has been recognized as a cause of focal paresis since the 19th century^16^ and duration of this Todd’s paresis can be quite short,^17^ mimicking a TIA. It was not clear in our patients whether the initial misdiagnosis was due to Todd’s paresis or general cerebral hypofunction. Similarly, the association of migraine with focal neurological symptoms has been well described.^18^ The association of encephalopathy and syncope with stroke mimics may be due to both inaccuracies in initial history taking or more likely, worsening of prior stroke deficits in this setting, as mentioned earlier. Finally, stroke survivors may present earlier than other patients because of increased awareness of the significance of stroke-like symptoms, thus increasing the rate of mimics in this group.

Our failure to propose a useful prediction model for the acute identification of stroke mimics, underscores the fact that clinical acumen is paramount in acute stroke care. The finding that almost all of the patients who had a final diagnosis that was non-stroke had a neurological diagnosis, demonstrates the need for early Neurological opinion. We must not miss this valuable opportunity to assess, diagnose and treat the high number of non-stroke patients presenting with an acute stroke syndrome.

Many patients with stroke or TIA are currently being diagnosed and treated by non-neurology trained physicians. In our view, this work presents compelling evidence for the involvement of stroke neurologists/stroke physicians in acute stroke care.

## Figures and Tables

**Table 1:** Breakdown of results.

Strokes	Stroke alert calls	Percentage	Stroke mimics	Stroke alert calls	Percentage
Transient Ischemic attack	36	23%	Encephalopathy	8	17%
Ischemic Stroke			Seizure	8	17%
Definite	94	59%	Syncope/Presyncope	7	15%
Probable	8	5%	Migraine	5	11%
Intracranial Hemorrhage			Functional	3	7%
Subarachnoid	3	2%	Dementia	3	7%
Subdural	4	3%	Vestibuloneuronitis/Labyrinthitis	3	7%
Parenchymal/Cerebellar	14	9%	Meningitis	1	2%
Venous Sinus Thrombosis	1	0.5%	Tumour	1	2%
			Unknown	5	11%
			Hip Fracture	1	2%
			Myoclonus	1	2%
**Total**	**160**	**100**%	**Total**	**46**	**100**%
